# Retinoids in the Pathogenesis and Treatment of Liver Diseases

**DOI:** 10.3390/nu14071456

**Published:** 2022-03-31

**Authors:** Marta Melis, Xiao-Han Tang, Steven E. Trasino, Lorraine J. Gudas

**Affiliations:** 1 Department of Pharmacology, Weill Cornell Medical College of Cornell University, New York, NY 10021, USA; mam2185@med.cornell.edu (M.M.); xit2001@med.cornell.edu (X.-H.T.); 2 Nutrition Program, Hunter College, City University of New York, New York, NY 10065, USA; st1647@hunter.cuny.edu

**Keywords:** nonalcoholic fatty liver disease, retinoic acid receptor, liver steatosis, vitamin A, hepatocellular carcinoma, alcohol-associated liver disease, retinoic acid

## Abstract

Vitamin A (VA), all-trans-retinol (ROL), and its analogs are collectively called retinoids. Acting through the retinoic acid receptors RARα, RARβ, and RARγ, all-trans-retinoic acid, an active metabolite of VA, is a potent regulator of numerous biological pathways, including embryonic and somatic cellular differentiation, immune functions, and energy metabolism. The liver is the primary organ for retinoid storage and metabolism in humans. For reasons that remain incompletely understood, a body of evidence shows that reductions in liver retinoids, aberrant retinoid metabolism, and reductions in RAR signaling are implicated in numerous diseases of the liver, including hepatocellular carcinoma, non-alcohol-associated fatty liver diseases, and alcohol-associated liver diseases. Conversely, restoration of retinoid signaling, pharmacological treatments with natural and synthetic retinoids, and newer agonists for specific RARs show promising benefits for treatment of a number of these liver diseases. Here we provide a comprehensive review of the literature demonstrating a role for retinoids in limiting the pathogenesis of these diseases and in the treatment of liver diseases.

## 1. Introduction to Retinoids, Vitamin A, and Retinoic Acid Receptors

### 1.1. Actions of the Retinoic Acid Receptors and Retinoic Acid (RA) in Transcriptional Activation

Vitamins, including vitamin A [1,2,3], vitamin B3 [4], and vitamin C [5] are capable, through various signaling pathways, of changing the epigenetic states of stem cells. This feature of some vitamins makes this area of research both timely and important for both long-term health and longevity, and for understanding and treating diverse diseases, such as cancer, heart disease, respiratory diseases, liver diseases, and Alzheimer’s Disease. In fact, in an analysis of a large cohort of men (>500,000 person-years of accumulated observation) over 3 decades, a statistically significant inverse association between higher serum vitamin A (retinol) and a lower risk of overall mortality was found [6].

The term ‘retinoids’ is used to designate all metabolites and chemicals with structures that are similar to that of the micronutrient vitamin A [7,8]. Vitamin A (all-trans-retinol) is an essential micronutrient that must be obtained from the diet. The absorption of vitamin A from the diet, the hepatic storage of vitamin A, and the mobilization of vitamin A from the liver have been recently reviewed by Blaner et al. [9]. Vitamin A is metabolized in various cell types to all-trans-retinoic acid (RA) [10], which is an endogenous agonist for the retinoic acid receptors (RARs) α, β, and γ [11].

**Figure 1:**
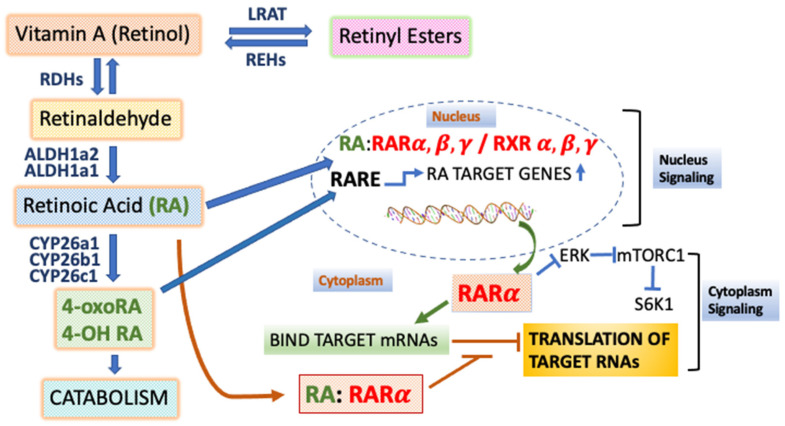
Model of Intracellular Retinol Metabolism and Molecular Actions. Retinoic acid (RA), the endogenous agonist for the three retinoic acid receptors (RARα, β, and γ), moves to the nucleus where it binds an RAR in a heterodimer with an RXR. This results in transcriptional activation of primary target genes that contain a retinoic acid response element (RARE) on DNA. A metabolite of RA, 4-OxoRA, can also act as an agonist for RARs. RARα can move to the cytoplasm, actively transported out of the nucleus, where RARα can regulate the rate of translation of target mRNAs into proteins, with different effects if the ligand RA is bound to RARα. RARα, without the ligand RA, can also inhibit ERK and mTORC1 signaling. The cytoplasmic actions of RARα have only been shown to date in neurons. Abbreviations: RDHs: retinol dehydrogenases; ALDH: aldehyde dehydrogenase; CYP26: Cytochrome P450 family 26 subfamily A member 1; ERK: extracellular signal-regulated kinase; mTORC1: mammalian target of rapamycin complex 1; LRAT: lecithin retinol acyltransferase; REH: retinyl ester hydrolase; S6K1: 40S ribosomal protein S6 kinase p70/p85.

The RARs are members of the larger nuclear receptor (NR) family, a family of DNA-binding proteins that acts to regulate transcription of distinct sets of target genes via binding to specific DNA sequences called ‘response elements’ at enhancers, defined as DNA sequences that increase transcription independently of their distance from a promoter [12]. The resulting changes in transcription lead to alterations in reproduction, metabolism, cell fate, and inflammation. RA response elements have been identified in many genes, such as Hoxa1 [13], HoxB1 [14,15,16,17], laminin B1 [18,19], and RARβ itself [20]. The RARs form heterodimers with the retinoid X receptors (RXRs) α, β, and γ to regulate the transcriptional activation of RAR target genes [21] (Figure 1).

The actions of these nuclear receptors can result in transcriptional activation or repression of sets of target genes. To activate transcription, ligand binding stabilizes the nuclear receptors in an active state in which they bind co-activator proteins that enzymatically make chromatin more accessible to RNA polymerase II [22,23]. Nuclear receptors are known to repress transcription by at least three different mechanisms. Without ligand binding, these NRs can bind co-repressor proteins that restrict the accessibility of chromatin for RNA polymerase II. Second, some nuclear receptors interact with ‘negative response elements.’ When bound to these ‘negative response elements’, these receptors adopt structures that favor co-repressor recruitment even in the presence of an agonist [24,25]. While these mechanisms are generally true for NRs, binding of RARs to ‘negative response elements’ has not been documented to date. A third mechanism is the repression by RA-bound RARs of the AP1 transcription factor complex consisting of Fos and Jun. Evidence suggests that RAR interferes with AP1 by binding to Jun and Fos (e.g., trans-binding) rather than by directly binding to a ‘DNA RA-response element’ [26,27].

Post-translational modifications of the RARs are also important for the proper activities of these RARs. Such modifications include the trimethylation modification at Lys347 of RARα [28] and phosphorylation of RARγ2 by p38MAPK [29]. These modifications impact the activities of the RARs.

The transcription of enhancer RNAs (eRNAs), small 1–2 kb transcripts, accompanies ligand-induced transcriptional activation, and these enhancer RNAs may act to mediate looping of enhancers and promoters [30,31]. Depending on the sets of enhancers affected by these eRNAs, eRNAs can repress target gene expression [32]. Estrogen receptor α, another member of the NR family, directly binds eRNAs via its DNA binding domain [32]. The roles eRNAs play in modulating the actions of RARs are not known.

Nucleotide excision repair occurs in response to DNA damage [33], but these DNA repair proteins are also involved in effecting efficient transcription. For example, even in the absence of genotoxic stress, the repair proteins XPF (ERCC4) and XPG (ERCC5) are recruited to the RARβ2 gene, which is transcriptionally activated by RA. If these proteins are knocked down in HeLa cells, the RA-associated activation of RARβ2 transcription is greatly reduced [34]. The endonuclease activities of these proteins, XPF and XPG, are required for chromatin looping and re-organization [34].

### 1.2. The Actions of RARs in Sequence-Specific Translational Control

RA/RARα can control the translation of specific mRNAs in neuronal dendrites, including the mRNA that encodes GluR1, the glutamate receptor subunit [35]. The activities of extracellular signal-related kinase (ERK) and mammalian target of rapamycin (mTOR) were elevated in neurons after RARα deletion, revealing a signaling pathway linking RARα to the neuronal-activity-dependent regulation of protein synthesis [36] (Figure 1). Moreover, RARα binds the RNA-binding protein, FMRP, and this binding is enhanced by the ligand, RA. This interaction between RARα and FMRP is required for proper *transcription-independent* RA signaling [37]. RARα transcription-independent actions to date have been demonstrated in neuronal cells, but future research may show such actions in other cell types. RARβ is also involved in the RA-dependent control of the rate of protein synthesis in hematopoietic stem cells [2].

### 1.3. Endogenous Ligands of the RARs

The endogenous agonist, RA, is metabolized to 4-oxo-RA and 4-OH-RA by the cytochrome P450 enzymes Cyp26a1 and Cyp26b1; 4-oxo- and 4-OH-RA have biological activity, but data show that these retinoids are generally further metabolized in a catabolic pathway that inactivates these ligands [38,39,40,41]. However, production of 4-oxo-RA by Cyp26b1 was recently shown to be required to maintain hematopoietic stem cells’ identity, and depletion of dietary vitamin A in animals resulted in a dysfunctional stemness phenotype in hematopoietic stem cells [42]. Notably, we showed that embryonic stem cells (ESCs) that lack Cyp26a1 differentiate poorly when compared to wild-type (wt) embryonic stem cells, even though the Cyp26a1 null ESCs contain much higher intracellular RA levels and express higher levels of one of the early, RAR-primary target genes, Hoxa1 [43,44]. We interpret our data to show that these more oxidized metabolites of RA are more effective agonists for regulating subsets of genes in stem cells and that the levels of these metabolites of RA play a major role in regulating stem cell differentiation [44]. Whereas Cyp26a1 knockout during development is lethal to the embryos [45], Cyp26a1 knockout in adult mice results in a relatively mild phenotype [46]. In contrast, Cyp26b1 knockout in adult mice reduces lifespan and causes systemic inflammation [47]. Whether this Cyp26b1 knockout phenotype is related to the dysfunctional stemness phenotype of hematopoietic stem cells described above is not clear at present, nor is it clear that these effects of Cyp26a1 or Cyp26b1 are mediated exclusively by their effect on retinoid metabolism.

Thus, various metabolites of RA, as well as RA itself and synthetic retinoid analogs, can bind to the RARs α, β, and γ, acting as agonists, to change cell fates and alter stem cell functions (Figure 1). Multiple cell fate outcomes are possible, given that there are three RARs and several RA metabolites that arise from the metabolism of RA by the enzymes Cyp26a1 and Cyp26b1.

## 2. Effects of Vitamin A Deficiency in the Liver

Many experiments have been performed to assess the effects of dietary vitamin A deficiency on liver functions. Adult, vitamin-A-deficient (VAD) rats showed hepatocyte vacuolization, a sign of steatosis, and mild inflammation in the liver [48]. In VAD rats, hepatic gluconeogenesis is decreased relative to that in vitamin-A-sufficient animals [49]. Moreover, a low level of retinoids in the liver at the time of carbon tetrachloride treatment greatly accelerated the process of liver fibrosis in rats [50]. This occurred concomitant with a selective loss of retinyl palmitate and a larger percentage of retinyl esters in the form of retinyl oleate and retinyl stearate. Furthermore, after common bile duct ligation, VAD rats displayed enhanced proliferation of bile duct epithelial cells [51].

Vitamin A is required for hepatocyte survival in a liver regeneration model partial hepatectomy (PHE) in rats [52]. Moreover, compared to wild-type mice, mice that lack lecithin:retinol acyltransferase (Lrat^−/−^) and thus are unable to store hepatic retinoids show delayed hepatic regeneration after partial hepatectomy [53]. These data indicate that a lack of hepatic retinoid hinders the survival and regeneration of hepatocytes after liver injury; however, the mechanisms by which low retinoids lead to defective hepatic regeneration were not identified in these studies.

## 3. RARs Are Required to Prevent Liver Disease (Steatohepatitis) and Hepatocellular Carcinoma in a Mouse Model

The RARs α, β, and γ and the RXRs α, β, and γ are all expressed in the liver [54]. The critical functions of the RARs in the liver were strikingly shown by using transgenic mice in which an RAR-α-dominant negative construct, driven by the albumin promoter, was expressed selectively in hepatocytes [55,56]. This construct suppresses the functions of all three RARs, α, β, and γ. The liver-selective RARα-dominant-negative mice showed both microvesicular steatosis at 4 months of age and a decrease in mitochondrial β-oxidation of fatty acids. These mice had hepatocellular carcinoma and adenoma of the liver at one year. Notably, feeding these mice a high-RA diet reversed these biochemical abnormalities and reduced the development of liver tumors. Thus, loss of RA actions specifically in the liver led to steatohepatitis and liver tumors. Since all three RARs were affected in these experiments, it is not possible to determine the roles of each of the RARs in the prevention of steatohepatitis and liver tumors. Conversely, treatment of wild-type mice with exogenously added RA shifted lipid metabolism toward reduced lipogenesis and increased catabolism [57,58]. We will discuss the actions of retinoids in the inhibition of non-alcohol-associated liver disease and liver cancer in rodent models and in humans in more detail in Section 4 and Section 6.

## 4. Non-Alcohol-Associated Fatty Liver Disease

Dr. Jürgen Ludwig first described nonalcoholic fatty liver disease (NAFLD) in 1980 [59]. NAFLD is a progressive disease, starting with hepatic steatosis; in some patients NAFLD progresses to nonalcoholic steatohepatitis (NASH), which is characterized by the development of fibrosis and cirrhosis [60]. Currently, NAFLD is the most common liver disorder in the world [61,62]. NASH will soon surpass alcoholic liver disease as the leading disease for liver transplant [63], and the United States spends more than $100 billion annually in direct medical costs primarily for NASH and its sequelae [64]. NAFLD is significantly associated with metabolic syndrome and obesity [64], and NAFLD increases the risk of type 2 diabetes and cardiovascular diseases [65]. Many NASH patients progress to liver cirrhosis, liver failure, and HCC [66]. The cumulative incidence of NAFLD-associated HCC was 7.6% in people who had had advanced fibrosis or cirrhosis for 5 years [67]. Currently, there are no FDA-approved pharmacological approaches for NAFLD, and therapy relies on the control of risk factors (diabetes, hypertension, obesity, and dyslipidemia) [68].

NAFLD pathophysiology is manifested by liver steatosis, i.e., lipid accumulation, fibrogenesis, and inflammation. Liver steatosis results in lipotoxicity that induces liver stress and injury, leading to fibrogenesis and inflammation [69]. In addition to this, dysbiosis, i.e., a disruption to the microbiota homeostasis in the gut, and dysregulated innate and adaptive immunity are crucial contributors to NAFLD progression [70]. Here, we will review the alterations in vitamin A metabolism and actions of retinoids in NAFLD with a focus on novel therapeutic approaches.

### 4.1. NAFLD Is Associated with Reductions in Hepatic Retinoids—A Possible Inverse Relationship between Intrahepatic Triglyceride Levels and Retinoids

For reasons that remain unclear, data show that hepatic retinoid levels are reduced in high-fat diet (HFD) and genetic murine models of NAFLD [71,72,73,74], human NAFLD [71,72,75,76], and acute liver injury [77]. The liver is the primary organ for retinoid storage, with approximately 80% of the total body retinoid pool stored as retinyl esters in triglyceride-rich lipid droplets in quiescent hepatic stellate cells (HSCs), specialized mesenchymal cells of the liver [78]. It is well documented that upon liver injury, HSCs transdifferentiate into activated myofibroblasts that execute wound repair, but in the process rapidly lose their retinoid content [78]. The loss of HSC retinoids in response to liver injury is incompletely understood, but it is reasonable to hypothesize that unchecked activation of HSCs is a key event in the NAFLD-associated hepatic retinoid reductions [79]. As such, evidence shows that hepatic retinoid levels are more severely depleted in advanced human liver disease (NASH vs. NAFLD) [75], and that hepatic retinoid levels show an inverse correlation with the severity of liver fat content and damage [71,76]. Trasino et al. [69] found that hepatic retinoid levels were inversely correlated with hepatic steatosis in murine and human NAFLD, suggesting that ectopic hepatic lipid itself may be an early trigger for the loss of hepatic retinoid before the onset of appreciable HSC activation. Recent evidence from a humanized mouse model of NAFLD supports this, as these mice do not develop liver scarring but show that hepatic retinoids are among the top analytes reduced in response to high-fat-diet induced NAFLD [74].

Nevertheless, it remains unclear if HSC loss of retinoid is a driver or a bystander of NAFLD progression [78]. Given emerging evidence of roles that hepatic lipids play in HSC biology [79] and a possible relationship between hepatic triglyceride levels and reductions in hepatic retinoids in NAFLD [71,76], in this section we will focus on current literature demonstrating a relationship between retinoids and hepatic triglyceride levels. This includes the primary pathways involved in regulating ectopic hepatic lipid accumulation: (i) de novo fatty acid synthesis, (ii) fatty acid oxidation, (iii) hepatic influx of plasma-free fatty acids (FFAs), and (iv) export of lipids from the liver in triglyceride-rich lipoproteins [68,69].

#### 4.1.1. De Novo Lipogenesis

Excessive free fatty acids in the liver are esterified into triglycerides that are stored as lipid droplets in hepatocytes, manifested as liver steatosis. De novo fatty acid synthesis converts non-lipid precursors into fatty acids, and de novo fatty acid synthesis can contribute almost 40% of intrahepatic triglycerides in subjects with NAFLD [80]. In mammalian cells, first, acetyl-CoA carboxylase converts acetyl-CoA to malonyl-CoA. Then fatty acid synthase (FASN), an enzyme containing multi-functional subunits with seven enzymatic activities: acetyl-CoA-ACP transacylase, malonyl-CoA-ACP transacylase, β-ketoacyl-ACP condensase, β-ketoacyl-ACP reductase, β-hydroxyacyl-ACP dehydratase, enoyl-ACP reductase, and palmitoyl-ACP thioesterase, initiates fatty acid synthesis using acetyl-CoA and malonyl-CoA [81]. The final product of FASN is palmitate, a saturated fatty acid. Fatty acids with longer chains and unsaturated fatty acids are produced from palmitate through different enzymes, i.e., elongases (ELOVLs) and desaturases [82,83]. Stearoyl-CoA desaturase 1 (SCD1) catalyzes the first desaturation reaction to produce the first double bond in palmitate and stearate [83]. Many lipid species, including triglycerides and phospholipids, are generated from saturated and unsaturated fatty acids.

**Figure 2:**
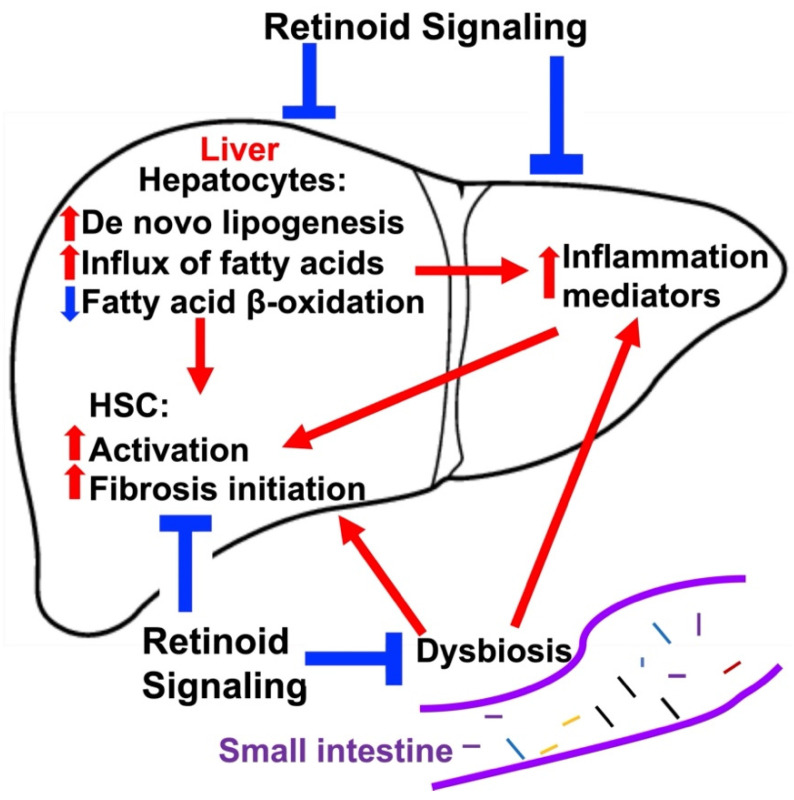
Retinoid actions in nonalcoholic fatty liver disease (NAFLD) and alcohol-related liver disease (ALD). De novo lipogenesis and free fatty acid influx contribute to liver steatosis manifested in both NAFLD and ALD. Liver-steatosis-induced lipotoxicity and dysbiosis in the gut results in hepatic inflammation and hepatic fibrosis. Retinoid signaling inhibits these events and attenuates these events, which can cause liver injury. Upward red arrows and downward blue arrow indicate increases and decreases, respectively. Abbreviations: HSC: hepatic stellate cell.

Retinoic acid suppresses lipid biosynthesis in mouse liver, and RA decreases the mRNA levels of both SREBF and FASN, which are involved in de novo lipogenesis [57,84] (Figure 2). RA also reduces lipid accumulation and steatosis in the liver of NAFLD mouse models [85,86,87,88].

We discovered that highly selective synthetic RARβ2 agonists reduce the mRNA and protein levels of multiple gene products (including SREBF, PPARG, and FASN) that are involved in lipogenesis in the liver in a high-fat-diet (HFD)-induced NAFLD mouse model [89,90] (Figure 2). Fructose and sucrose intake promotes de novo lipogenesis in the liver [91,92], and a recent pre-clinical study indicates that a fructokinase (also called ketohexokinase) inhibitor improves NAFLD/NASH [91]. We discovered that, in a high-fat diet mouse NAFLD model, one of the key mechanisms by which a highly selective synthetic RARβ2 agonist suppresses lipid accumulation in the liver could be inhibition of fructose metabolism [90] (Figure 2).

#### 4.1.2. Fatty Acid Oxidation

In addition to suppressing de novo lipogenesis, RA treatment upregulates hepatic lipid oxidation by increasing the expression of PPARα, FGF21, CPT1, and UCP2 [57,72,93]. Rdh10^+/−^ mice, which have reductions to hepatic RA levels, develop increases in hepatic triglycerides and reductions in expression of genes involved in fatty acid β-oxidation, including PPARα [87]. It is unclear if each of the RARs is dedicated to regulating specific aspects of fatty acid β-oxidation, or if there is redundancy among them. The data currently suggest roles for both RARα and RARβ in regulating hepatic β-oxidation of fatty acids. For example, as mentioned in Section 3 above, mice expressing a hepatocyte-specific dominant negative RARα that suppresses the signaling of all endogenous RARs develop hepatic steatosis, with impaired capacity for mitochondrial β-oxidation of fatty acids, which can be reversed with RA treatment [56]. Interestingly, these mice also show increased hepatic expression of genes involved in peroxisomal β-oxidation of lipids [56], demonstrating that the role of RARs in oxidation of fatty acid is more complex and incompletely understood. A role for RARβ in hepatic fatty acid oxidation has also been demonstrated, as mice overexpressing RARβ in the liver show increases in FGF21-mediated fatty acid β-oxidation and an increase in whole-body energy expenditure [93]. In line with this, RARβ2 selective agonists increase the transcript levels of genes that promote and mediate mitochondrial fatty acid β-oxidation, including PPARα and CPT1A [89]. In contrast to the effects of RARβ2 selective agonists on liver steatosis in HFD-induced NAFLD models [89,90,94], the RARα agonist Am 80 exacerbates [95] and the RARγ agonist CD1530 [94] has no major effect on liver steatosis in these models, respectively. Although murine genetic studies demonstrate a role for RARα and RARβ in the regulation of fatty acid oxidation in the liver [56,93], studies using RAR-specific agonists can also further enhance understanding of the effects of specific RAR activation in mediating hepatic fatty acid oxidation.

#### 4.1.3. Free Fatty Acid Influx to the Liver and Export of Lipids from the Liver

Another primary source of fatty acids in the liver is from the influx of free fatty acids from blood [69], including oleate and palmitate [96]. There have been few studies of the impact of natural retinoids on hepatic influx of free fatty acids; however, we found that a RARβ2-selective agonist inhibits high-fat-diet induced increases in the mRNA and protein levels of the fatty acid transporter CD36, indicating that this agonist attenuates free fatty acid influx to the liver [90] (Figure 2). To date, we found no studies reporting the effects of retinoids on lipid export from the liver.

### 4.2. Targeting Hepatic Stellate Cell (HSC) Activation and Fibrogenesis

Fibrogenesis during NAFLD progression is initiated by activation of quiescent hepatic stellate cells (HSCs) that produce excessive extracellular matrix proteins, generating fibrous scars [97]. HSCs and fibrosis are the targets for novel therapy development for NAFLD, including using α-bromomethylene phosphonate lysophosphatidic acid and silencing the lysophosphatidic-acid-producing enzyme autotaxin to inhibit activated HSCs, tyrosine kinase inhibitors (sorafenib, imatinib, or nilotinib) to suppress HSC proliferation and migration, and lysyl oxidase inhibitors to reduce ECM deposition [98].

The effects of retinoids on HSCs remain controversial [99]. RA and retinol mitigate carbon tetrachloride (CCL4)-induced liver fibrosis in mice [98] and cholestatic liver fibrosis in rats [100], respectively. On the contrary, another study suggests that RA in HSCs promotes HSC activation and liver fibrosis in rats [101]. An isomer of all-trans RA, 9-cis retinoic acid, promotes liver fibrosis in rats [102]. Treatment with 9-cisRA also caused resistance to IFN-γ therapy in advanced stages of liver fibrosis in mice [103]. Some studies have suggested that the varied effects of retinoids on HSCs are because of different responses of HSCs to different retinoid species, such as RA and 9-cisRA, which may selectively activate different RARs [104]. Indeed, RARα activation promotes inflammatory signaling and fibrosis in lipopolysaccharide-activated HSCs [105]. RARα activation also promotes HSC activation in a high-fat-diet (HFD)-induced NAFLD mouse model [95]. Acting through RARβ and RXRα, RA reduces type I collagen production in in vitro cultures of activated HSCs through RARβ and RXRα [106]. A selective RARβ2 agonist mitigates HSC activation and early fibrosis events in HFD-induced NAFLD mouse models [90,94], while RARγ activation has no effect [94]. In addition to the direct effects of retinoids on HSCs, some studies suggest that retinoids modulate the interaction between HSCs and liver natural killer (NK) cells [107] that kill activated HSCs and mitigate liver fibrosis [108,109]. However, these studies did not explore which RAR(s) is (are) involved in these actions. We conclude that additional studies using genetics or selective agonists are needed to resolve the roles of retinoids in promoting and/or limiting fibrosis (Figure 2).

### 4.3. Targeting Inflammation in NAFLD

Intermittent, chronic, sterile low-grade inflammation (metaflammation) in the liver occurs in 10–30% of NAFLD patients [70,110,111]. Gut microbiome, dietary factors, and certain lipid species contribute to this metaflammation in the liver in NAFLD [70]. Both innate and adaptive immune systems are involved in this process [69,70]. Natural and synthetic retinoids suppress the liver’s resident macrophages’ production of inflammation mediators TNFa [112] and IL12 [113]. We discovered that a selective RARβ2 agonist attenuates the increases in the expression of pro-inflammatory mediators in NAFLD mouse models, including TNFα, CCL2, CCR2, and IL1β, while a RARγ agonist has no effect [90,94] (Figure 2). Interestingly, RARα activation exacerbates inflammation, indicated by F4/80 staining, in an HFD-induced NAFLD mouse model [95]. These data are consistent with earlier studies in that the three RARs have different and sometimes antagonistic effects, likely because of competition at some DNA response elements [114], i.e., RARγ1 can inhibit other RARs’ actions via competition for the response element and direct interaction with other receptors in model cell culture systems.

### 4.4. Targeting the Effects of the Gut Microbiome on NAFLD Progression

Compelling evidence shows that the gut microbiome-liver axis plays an important role in NAFLD progression [115]. Gut bacteria modulate the gut-liver axis via intestinal FXR-FGF19 signaling that regulates bile acid synthesis and lipid and glucose metabolism [69]. Additionally, dysbiosis in the gut, i.e., an alternation in the gut microbiota homeostasis, results in an increase in intestinal permeability, leading to invasion of gut bacteria and their products in the liver that trigger downstream inflammatory responses and HSC activation, resulting in worsening of liver injuries in NAFLD [116,117]. Although the exact mechanism is not understood, one study suggests that RA may prevent dysbiosis in the gut [118] (Figure 2). RA also reduced intestinal permeability by improving the intestinal barrier function, and this effect is mediated via RARβ [119]. Thus, RARβ may exert an effect on NAFLD progression via its actions in the intestine, and RARβ and its signaling pathway in the gut could be novel targets for NAFLD therapy development.

## 5. The Roles of Retinoids in the Pathogenesis and Treatment of Alcohol-Associated Liver Disease (ALD)

Retinoids have been extensively studied in ALD, both in rodent models and in human disease. Here we review much of this literature.

### 5.1. Chronic Alcohol Abuse Is Associated with Depletion of Liver Retinoids

Alcohol-associated liver disease (ALD) is one of the most common causes of liver cirrhosis and is responsible for approximately 25% of all liver-related deaths globally [120]. ALD has a broad clinical spectrum; beginning with simple steatosis, ALD can progress to alcoholic steatohepatitis, alcoholic cirrhosis, and end-stage liver disease [121]. The liver is responsible for the detoxification of alcohol [121], but it is also the primary organ for the metabolism and storage of retinoids [122], with approximately 80% of the total body retinoid pool stored in the liver HSCs [78]. There are a number of enzymes shared both by alcohol and retinoid metabolism in the liver [123,124,125], and over more than 50 years, researchers have found abnormal retinoid homeostasis in individuals suffering from chronic alcoholism and ALD [126,127,128,129,130,131]. Notably, even prenatal alcohol exposure results in a major decrease in retinyl ester levels in the livers and lungs of adult rodents [132]. Increases in extra-hepatic tissue RA [133] also indicate long-term effects of prenatal alcohol exposure on whole-body retinoid homeostasis.

A landmark study by Leo et al. [128] demonstrated that, when compared to livers from normal control subjects, levels of total hepatic retinoid content (retinyl esters and retinol) were markedly decreased with increasing severity of ALD (from simple steatosis to steatohepatitis). Moreover, subjects with early stages of ALD (simple steatosis) had normal serum retinol, and only in subjects with advanced stages of ALD (alcoholic steatohepatitis and cirrhosis) were serum retinol levels depressed compared to controls [128]. Other human studies have similarly reported hepatic retinoid reductions in individuals with chronic alcoholism [134] and serum retinol levels that are unchanged with chronic alcohol abuse [135,136], but depressed in advanced ALD [130]. The implications of these findings are that individuals in early stages of chronic alcoholism who present with normal serum retinol levels may already have severely depleted hepatic retinoid levels.

What remains unclear is whether depletion of hepatic retinoid itself promotes liver damage and the onset and progression of ALD [123]. It has been hypothesized that retinoid deficiency may be involved in the keratinization of the liver and formation of Mallory Bodies [137], hepatic lesions associated with alcoholic steatohepatitis and cirrhosis [138]. However histopathology studies show that the prevalence of Mallory Bodies in individuals with and without hepatic retinoid depletion is similar [128]. Clugston et al. [139] reported that alcohol-fed LRAT^−/−^ mice, which are unable to store hepatic retinoid, are protected against alcohol-mediated increases in extra-hepatic tissue retinoid levels. However, no assessment of liver damage and pathology in alcohol-fed LRAT^−/−^ mice versus alcohol-fed wild-type mice was reported [139]. Evidence does show that LRAT^−/−^ mice are not more prone to bile duct ligation or chemically induced fibrotic liver disease, though they are protected against chemically induced hepatocarcinoma [140]. Given the convincing body of data showing that chronic alcohol abuse leads to reductions in hepatic retinoids [126,127,128,129,130,131], more studies are needed to determine whether reductions in hepatic retinoids are specifically involved in the progression of ALD.

### 5.2. Mechanisms of Hepatic Retinoid Depletion in ALD

Numerous factors might contribute to the reductions in hepatic retinoid in chronic alcohol abuse and ALD, including poor dietary retinoid intake, poor absorption, and increased retinoid demands from infection [131,141]. However, alcohol feeding studies in vivo demonstrated that even in the absence of any dietary retinoid, hepatic alcohol metabolism itself promoted reductions in hepatic retinoid content [129,142,143]. Based on the understanding that liver retinol is primarily derived from hepatocytes, and retinyl esters from HSCs [78], it is likely that alcohol promotes reductions in retinoids in both these cell types.

Since the findings by Leo et al. [128], numerous in vitro and in vivo studies have focused on three potential mechanisms for alcohol-mediated reductions to hepatic retinoid: (i) increased retinoid catabolism, (ii) decreased all-trans retinoic acid (RA) synthesis, and (iii) increased hepatic retinoid mobilization [123]. The increased catabolism theory purports that chronic alcohol intake increases numerous xenobiotic enzymes, including cytochrome P450 2E1(CYP2E1), the major CYP450 in the microsomal ethanol-oxidizing system (MEOS) [144], which promiscuously catabolizes and reduces hepatic retinoids (i.e., retinol, retinyl esters, and RA) [143,145]. For example, Liu et al. [143] demonstrated that incubation of liver microsomal fractions high in CYP2E1 from alcohol-treated rats resulted in increased catabolism of RA and appearance of RA catabolites, such as 4-oxo-RA. In another study, Liu et al. [145] reported that alcohol-fed rats treated with the CYP2E1 inhibitor, chlormethiazole, had higher hepatic retinol and retinyl-palmitate compared to untreated alcohol-fed rats, suggesting that CYP2E1 is a key CYP450 enzyme involved in the hepatic degradation of all retinoid species found in the liver. That notion, however, was challenged in a study by Clugston et al. [139] that demonstrated that Cyp2e1^−/−^ mice were only protected from alcohol-mediated reductions in hepatic retinol, not retinyl esters [139]. The Clugston et al. study [139], and a similar study by Ferdouse et al. using alcohol-treated Cyp2e1^−/−^ mice [146], did not determine if loss of CYP2E1 could mitigate alcohol-driven reductions in hepatic RA.

The Ferdouse et al. study [146] did show, in agreement with a recent RNA-sequencing study of livers from alcohol-treated mice by Melis et al. [147], that 3–4 weeks of chronic alcohol treatment leads to robust and broad hepatic mRNA increases in numerous CYPs (including Cyp2c29, Cyp3a11, Cyp26a1, and Cyp26b1) that are capable of degrading retinoids [125]. These data strongly suggest that in addition to CYP2E1, alcohol-induced CYP450s, including the RA hydroxylases CYP26A1 and CYP26B1, are likely involved in alcohol-mediated reductions in hepatic retinoids.

The less explored ‘decreased RA synthesis hypothesis’ suggests that chronic alcohol intake can potentially competitively inhibit the synthesis of RA from retinol, due to the overlap of enzymes involved in the two-step oxidative metabolism of alcohol to acetate and retinol to RA (i.e., alcohol/retinol dehydrogenase 1 (ADH1), and aldehyde/retinaldehyde dehydrogenase A1 (ALDH1A1), reviewed in [148]). However, there is little convincing data that the overlap between alcohol and retinol oxidation/metabolism has any physiological effects on retinoid homeostasis [148]. Furthermore, there is debate as to whether alcohol leads to reductions to hepatic RA [123,148]. Three studies using highly sensitive tandem mass spectrometry (LC-MS/MS) approaches to measure hepatic RA in male mice show that chronic alcohol feeding resulted in either no changes [133,147] or reductions in hepatic RA levels [146]. The discrepancies in these findings may be partly explained by data from Kane et al. [133], who performed the most comprehensive examination of both acute and chronic effects of alcohol on hepatic and extra-hepatic RA. They showed that alcohol intake causes rapid increases in hepatic RA synthesis, followed by equally rapid metabolism, likely by hepatic CYP26 RA hydroxylases and other CYPs, resulting in no long-term changes to steady-state hepatic RA levels. Therefore, the data from Kane et al. [133] suggest that the timing of the hepatic RA measurements is likely critically important in the determination of the effects of alcohol on hepatic RA levels. In the same regard, differences in the length of reported alcohol exposure and dose across these studies (4 weeks of 6.5% *v*/*v* in Kane et al. [133], vs. 3 weeks of 5% *v*/*v* in Melis et al. [147], vs. 2 weeks of 6.4% *v*/*v* in Ferdouse et al. [146]), should also be considered when comparing these findings.

Nevertheless, the conclusions from these studies were in agreement that alcohol promotes hepatic RA catabolism [133,146,147]. However, this phenomenon does not appear to occur in some extra-hepatic tissues, as Kane et al. [133] also demonstrated that chronic alcohol exposure increases RA levels in the testes, and, consistent with another report [149], the brain. Kane et al. [133] also found that hippocampal mRNA levels of CYP26 hydroxylases and other RA-metabolizing CYPs, such as CYP2C39 [125], were unchanged, suggesting that tissues which lack sufficient RA-metabolizing enzymes are susceptible to RA toxicity in response to chronic alcohol intake. Given the challenges in measuring endogenous RA (i.e., low tissue levels and rapid metabolism), future studies of the effects of alcohol on hepatic and extra-hepatic RA levels should seek to standardize LC-MS/MS analytical approaches of endogenous RA levels, which have been addressed in numerous methodology papers [150,151,152].

The increased ‘hepatic retinoid mobilization’ theory was already put forward in the first observations by Leo et al. [128] of hepatic retinoid depletions in human subjects with ALD. This theory is reasonable, given that the liver is the primary organ for retinoid export to maintain steady-state serum retinoid levels [122,153]. The first evidence of increased hepatic mobilization of retinoids came from a study by Kane et al. [133], which demonstrated that both acute and chronic alcohol consumption resulted in increases in RA levels in extrahepatic tissues, including the serum, brain and testis. The increases in serum RA were accompanied by reductions in hepatic retinoids and high levels of retinol and retinoid dehydrogenases in extra-hepatic tissues [133], suggesting that the liver was the likely source. In a study by Clugston et al. [139], it was shown that with chronic alcohol abuse there is a two-phase hepatic response to alcohol; first, there is an increased hepatic retinoid export to serum and extra-hepatic tissues, followed by a phase of increased hepatic degradation, which was subsequently demonstrated to likely be due to marked rises in CYP26A1, CYP26B1, and other CYPs capable of degrading retinoids [146,147]. Using alcohol-fed LRAT^−/−^ and cellular retinol binding protein 1^−/−^ (RBP1) mice, Clugston et al. [139] convincingly demonstrated that extra-hepatic tissue retinol is derived from the liver during chronic alcohol intake and that RBP1 plays a key role in extra-hepatic accumulation of excessive retinoids. In another model system, alcohol stimulated the differentiation of embryonic stem cells by increasing the influx and metabolism of retinol, which then led to RARγ-dependent transcription of RBP1 and other genes involved in RA synthesis [154].

### 5.3. Effects of Retinoids on ALD

As ALD progresses, the severity of the reductions in hepatic retinoids also increases [128], suggesting that loss of hepatic retinoid content may be involved in the progression of ALD. In keeping with the hypothesis that endogenous retinoids are hepatoprotective against ALD, many studies have examined the protective properties of exogenous retinoids (including RA and retinyl-palmitate) [131,155,156,157,158,159,160,161]. A study by Motomura et al. [155] demonstrated that 16 h of ex vivo RA (500 nm) treatment of hepatic Kupffer cells (macrophages), isolated from rats fed alcohol, reduced lipopolysaccharide (LPS)-mediated expression of a number of pro-inflammatory cytokines, including TNF-α, IL-6, IL-1α, and IL-1β (Figure 2). Interestingly, this study found that RA levels were lower in Kupffer cells from alcohol-fed rats compared to controls, suggesting that RA reduction in Kupfer cells may be associated with progression of ALD. Similarly, a study by Chung et al. [156] showed that alcohol-fed rats treated with RA for 30 days resulted in restoration of endogenous RA and retinol levels, but not retinyl palmitate, in the liver; mitigation of hepatocyte proliferation; and expression of the cell cycle regulatory proteins c-Jun and cyclin D1. In a follow-up study, Chung et al. [157] demonstrated that 6 months of exogenous RA treatments in alcohol-treated rats mitigated changes in hepatic protein levels of the cell cycle regulatory proteins phosphorylated Jun N-terminal kinase (JNK) and its upstream regulator mitogen-activated protein kinase kinase-4 (MKK-4). Interestingly, despite evidence that alcohol promotes apoptosis of hepatocytes [162], Chung et al. [157] found that alcohol suppressed hepatocyte apoptosis, which was increased by RA in alcohol-fed rats [157]. The findings by Chung et al. [156,157] are consistent with the established anti-proliferative, anti-cancer properties of RA and other retinoids [7], suggesting that with long-term (>3 months) chronic alcohol intake, exogenous RA may increase apoptosis of severely damaged hepatocytes, reducing the risk of hepatocellular carcinoma that is associated with ALD [163].

In a more comprehensive analysis of the biochemical and histopathological hallmarks of ALD, Pan et al. [158] showed that a daily RA dose of 150 μg/kg mitigated hepatic steatosis and liver damage, as measured by reductions to serum aspartate aminotransferase (AST) and alanine aminotransferase (ALT), in rats fed alcohol [158]. Unlike low dose RA, which restored hepatic RA and retinol, a higher dose of RA also restored hepatic retinyl esters in alcohol-fed rats [158]. The latter result is consistent with evidence that RA itself can increase retinol esterification in the liver [164]. Thus, high doses of RA could be a pharmacological approach for preserving hepatic retinoid stores with chronic alcohol abuse. Given that they found no differences in the liver protection between the lower and higher doses of RA, retinol, rather than retinyl esters, may be involved in the hepatoprotective properties of exogenous RA.

Many potential mechanisms of the anti-ALD properties of RA remain unexplored, but, as discussed here, the hepatoprotective effects of RA in models of ALD are consistent with a large body of evidence that RA favorably modulates ALD-relevant pathways, including hepatic lipid metabolism [165], oxidative stress, and inflammation in other fibrotic liver diseases, such as NAFLD [166,167,168,169] (Figure 2).

These studies show promise for RA as a potential anti-ALD drug, and they suggest that RA would have to be consumed concomitantly with alcohol as a prophylactic therapy against the onset of ALD. However, long-term RA therapy in ALD would be challenging, given the rapid, first-pass metabolism of RA [164]. For example, pharmacokinetic data show that patients given continuous, daily oral RA therapy for leukemia show decreases in systemic RA levels and in some cases, returns to baseline levels after approximately 21 days [170,171]. Moreover, in individuals who struggle with alcohol cessation, long-term RA therapy would be further hampered given that alcohol-mediated increases in CYP2E1 and other xenobiotic CYP450s increase the catabolism of RA and other retinoids [143,145,146]. Supplementation with retinyl palmitate or the pro-retinoid β-carotene to raise endogenous RA levels may not be feasible or safe, because, for reasons that remain unclear, supplementation with these RA precursors in the presence of alcohol promotes liver damage, myofibroblast formation, and fibrosis in rat models of ALD [131,159,160,161]. A possible alternative approach may be the use of oral RA treatment in conjunction with a chemical CYP2E1 inhibitor, which can prevent RA catabolism and protect against ALD [145]. Notably, a new class of CYP2E1 inhibitor has been used for prophylactic treatment of early stages of ALD [172].

Another novel approach for the treatment or prevention of ALD could be the use of synthetic retinoids that might not be as negatively impacted by alcohol as natural retinoids. Melis et al. [147] reported that in mice concomitantly treated with alcohol (5% *v*/*v*) and an orally available, synthetic agonist of retinoic acid receptor β2 (RARβ2), AC261066 [173], liver damage and the clinical pathology associated with ALD were decreased, including reductions in liver triglycerides, micro- and macrovesicular steatosis, oxidative stress, and serum AST and ALT levels (Figure 2). That study also reported that despite marked reductions in hepatic retinoids in the alcohol-fed mice, liver levels of AC261066 were unaffected by alcohol treatments [147]. Unlike the reports with RA treatments [156,158], AC261066 did not restore the alcohol-driven reductions in hepatic retinol or retinyl esters, and hepatic RA was unchanged between control and alcohol plus AC261066 treatment groups. Whether the hepatoprotective effects of AC261066 involved RARβ2 signaling was not rigorously tested. Interestingly, alcohol treatments still increased hepatic mRNA levels of *RARβ2* and other RAR target genes, including *Cyp26a1*, *Cyp26b1,* and *LRAT* [147].

The synthetic retinoid fenretinide (*N*-(4-hydroxyphenyl)retinamide; 4-HPR),which is known for its anti-cancer effects [174], also has anti-ALD properties. A study by Tang et al. [175] reported that mice chronically fed alcohol for 3 weeks and treated with 4-HPR had reductions in alcohol-associated steatosis, oxidative stress, and liver damage. Daily 4-HPR treatments also diminished alcohol-mediated gut damage and systemic endotoxemia [175], which is a critical aspect of the molecular pathogenesis of ALD and alcohol-associated systemic damage [121]. Tang et al. [175] found that the hepatic mRNA levels of the retinoid target genes *Cyp26a1* and *RARβ2* [153] were unchanged by the 4-HPR treatments, which is consistent with the fact that 4-HPR is an atypical retinoid, in that it also possess biological properties that are RAR-independent [176].

Collectively, these data suggest a role for exogenous retinoids in the prevention and treatment of ALD. However, further studies are needed to identify whether long-term retinoid treatment is feasible, given the effects of alcohol on retinoid metabolism. Alternative approaches, using either synthetic retinoids or high-affinity RAR agonists that show hepatoprotective properties but are less susceptible to the metabolic changes induced by alcohol, should also be given consideration as novel approaches for retinoid treatment of ALD.

## 6. Retinoids in Liver Cancer

Liver cancer is the third most deadly cancer worldwide following lung and colorectal cancers [177,178]. The most prevalent form of liver cancer is hepatocellular carcinoma (HCC), which mainly develops in individuals with chronic hepatitis B and C (HBV and HCV) or with alcohol-related chronic liver disease [177]. Additionally, because of the global increases in NAFLD/NASH, obesity, and metabolic syndrome, all of which are associated with HCC, global HCC cases are expected to increase in the next decade [179]. Despite the increased precision in the diagnosis of HCC, this tumor is usually recognized in advanced stages and treatments are still not effective. Therefore, HCC has a poor outcome, with one of the highest rates of recurrence and the lowest 5-year survival rates of 5–10% [180].

In patients with HCV-associated, chronic liver disease there is a progressive decrease in serum retinol levels as the disease becomes more severe [181]. However, the potential roles of retinoids in the prevention and treatment of HCC are not fully understood, and this lack of understanding contributes to our limited ability to design effective therapies for this type of tumor. Here we discuss some recent findings that shed light on mechanisms linking RA signaling to HCC prevention and therapy, opening new avenues for the potential uses of retinoids in HCC treatment.

### 6.1. Association of Hepatitis B and C Viruses with Abnormal RARβ Function

Despite both viruses being hepatotropic, HBV and HCV cause HCC via distinct pathways. The HBV genome has a partially double-stranded DNA genome that gives origin to multiple proteins essential for the virus life cycle. The gene encoding the HBx protein is associated with direct carcinogenic potential, in part because of its ability to integrate into the host genome, its ability to interact with the host cell cycle, transcription factors, and DNA repair, hence, the name ’viral oncoprotein’ [182]. HCV has a single-stranded RNA genome whose ability to induce carcinogenesis is primarily by promoting accumulation of lipids in the liver via increasing endoplasmic reticulum stress [183,184], and by eliciting a dramatic immune response in the liver [185]. People with HCV often have lower serum vitamin A than healthy controls [186], suggesting two key scenarios in HCV pathogenesis, i.e., loss of vitamin A may increase susceptibility to HCV infection and it may promote hepatic fibrosis because of the vitamin A depletion in hepatic stellate cells, which store vitamin A in healthy individuals. These aspects are discussed in detail in a separate review [187]. In support of these observations, vitamin A showed activity against HCV infection in cell culture models [188,189,190]. Despite the fact that this antiviral effect was also observed in HCV-infected patients after treatment with RA alone or in combination with standard HCV therapies, the patients experienced viremia after the treatment [191].

Among the RARs, there may be a unique relationship between RARβ and HBV. Indeed, the gene for RARβ was first discovered in human HCC, where it flanks an HBV integration site [192,193]. In the case of human HCC, HBV integration often causes a microdeletion and rearrangement in the RARβ open reading frame which produces an HBV-RARβ chimera that possesses oncogenic properties, suggesting that aberrations in RARβ may be involved in HBV-associated HCC [194]. There is evidence showing that HBx transfection into HepG2 cells causes a decrease in RARβ2, the most abundant RARβ isotype, by an HBx-driven aberrant promoter methylation. Consequently, growth arrest of these HBx-transfected HepG2 cells upon RA treatment does not occur [195].

**Figure 3:**
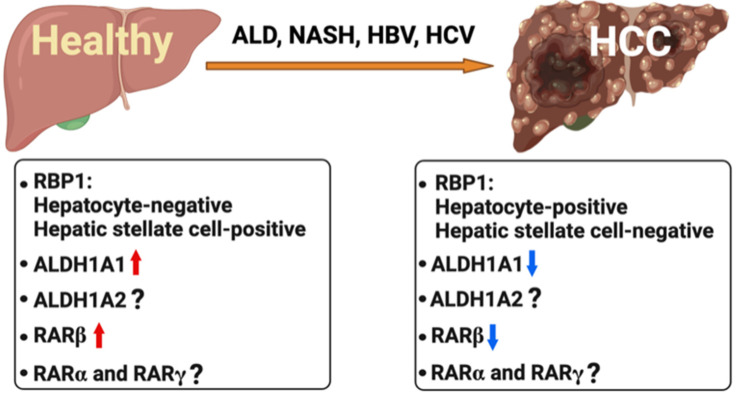
Retinoids and hepatocellular carcinoma (HCC). Alcohol-related liver disease (ALD), non-alcoholic steatohepatitis (NASH), and hepatitis virus B and C infection (HBV and HCV) predisposes to the development of HCC. While there is strong evidence that in healthy liver RBP1 (retinol binding protein 1) is expressed predominantly in hepatic stellate cells rather than in the hepatocytes, in HCC RBP1 expression is lost in hepatic stellate cells and observed in hepatocytes. ALDH1A1, which is one of the enzymes responsible for the conversion of retinaldehyde to retinoic acid, is lower (blue downward arrows) in HCC than in healthy liver (red upward arrows). Lower RARβ levels compared with adjacent non-tumor and healthy livers also characterize HCC. There is limited research on RARα and γ in HCC.

Although other studies showed no differences in RARβ expression between HCC and non-tumor tissues [196], more recent studies confirmed the decrease in vitamin A in human HCC associated with HCV infection [181], as well as a reduction in RARβ in cirrhosis and HCCs of unknown etiology compared with normal livers [197,198] (Figure 3).

### 6.2. Aberrant Regulation of Retinoid Metabolism and RARβ in Hepatocellular Carcinoma

Retinoids modulate numerous cellular functions, including signal transduction, cell proliferation, apoptosis, and immunity [122,199], and it is likely that endogenous retinoids may also play a role in the inhibition of carcinogenesis. As discussed in Section 3 above, in mice deficient in all three RARs (α, β, γ) because of the expression of a dominant negative RAR construct expressed only in the liver, liver steatosis was seen at 4 months of age and HCC developed after 12 months [56]. Treating these mice with exogenous RA prevented the appearance of steatosis and its progression to cirrhosis (8). In rodent models of alcohol-associated liver cancer, there is a major reduction in endogenous retinoids [200,201].

Additional evidence of RAR dysfunction in HCC comes from the comparison across various liver conditions, including liver regeneration, multiple fetal development stages, human hepatocellular carcinoma, hepatoblastoma cell lines (i.e., HepG2), and finally, less frequent types of liver cancer, such as adenoma, fibrolamellar carcinoma, and cholangiocarcinoma. The levels of RARβ mRNA in this spectrum of liver diseases ranged from low to undetectable in all liver cancers and other conditions, except for cholangiocarcinoma [202]. Moreover, Cortes et al. demonstrated that decreased RARβ activity, generated by treating cultured hepatic stellate cells with a RARβ antagonist (the drug used in this study targets both RARβ and γ), resulted in an increase in myosin light chain 2 (MLC-2), a protein produced by hepatic stellate cells that confers cell contractility and invasive potential [197]. Thus, a reduction in RARβ level and/or activity may be one driver of liver carcinogenesis.

One of the key biomarkers to assess HCC presence is alpha-fetoprotein (AFP) [203,204,205]. Some have proposed a mechanism in which AFP acts as a suppressor of RARβ and γ, as shown by co-immunoprecipitation experiments [206,207]. Although the potential mechanisms behind this interaction could be crucial for our understanding of the RA signaling pathway’s role in limiting HCC, there is the need to better define whether AFP binds to or acts on specific RARs.

While the RARs exert transcriptional control of the retinoid signaling pathway, enzymes that synthesize RA may play major roles in liver homeostasis and in the pathophysiology of liver cancer. One of the enzymes that catalyzes the conversion of retinol to retinoic acid, ALDH1A1 [208], showed a negative correlation with HCC recurrence in patients that underwent HCC-related liver transplantation [209]. Likewise, RBP1, which is responsible for the transport of intracellular retinol, exhibited the classic cytoplasmic expression in hepatic stellate cells and myofibroblasts in healthy human liver, whereas in human HCC RBP1 staining was aberrantly distributed in the cytoplasm and nucleus of neoplastic hepatocytes as well as in a few myofibroblasts in non-tumor liver tissue while RBP1 expression was almost absent in hepatic stellate cells [210]. These findings are recapitulated in Figure 3. It is not clear if RBP1 expression in hepatocytes occurs as a compensatory effect from the loss of RBP1 expression in hepatic stellate cells or because RBP1 in hepatocytes participates in different pathways. The mechanisms behind these findings and the potential crosstalk between hepatic stellate cells and hepatocytes are presently unknown.

### 6.3. Therapeutic Potential of Retinoids in HCC

Despite the finding that RA treatment caused growth arrest in the hepatoma HepG2 cell line [211] and decreased the level of proline isomerase 1 (PIN2), one of the proteins highly expressed in HCC [212], the use of RA as a therapeutic option in HCC treatment requires further research. One important approach might be to use isoform-specific agonists, and we outline newer synthetic retinoids that may overcome the limitations of the rapid metabolism of RA and the binding of RA to all of the RARs, while preserving the anti-cancer properties of retinoids such as RA. This approach is similar to that described in Section 1 and Section 5.

Fenretinide is a compound with RAR-dependent and RAR-independent mechanisms of action [176]. For example, fenretinide induced apoptosis and showed anti-proliferative effects in HepG2, Huh 7, and HepB3 cells through RARβ actions [213,214,215,216]. Recently, researchers reported that sulfarotene (WYC-209), an acyclic retinoid, overcame HCC resistance in a subset of cancer cells that were responsible for drug resistance (i.e., tumor-repopulating cells), possibly via RARα [217]. A promising retinoid at present for the prevention of HCC recurrence is peretinoin (NIK-333) [218,219], which is in clinical trials that are discussed in a separate review [220]. Pre-clinical studies showed that peretinoin suppressed steatosis and tumorigenesis in a mouse model of diet-induced NASH and HCC by promoting autophagy and inhibiting pro-inflammatory pathways [221]. Peretinoin also resulted in a reduction in activated hepatic stellate cells and oval cells in rats [222]. Peretinoin’s mechanism of action involves the canonical RA-signaling pathway via increases in the RAR and RXRs in multiple experimental models [223,224]. Another synthetic retinoid, 4-amino-2-trifluoromethyl-phenyl retinate (ATPR), inhibited cell proliferation and caused apoptosis more efficiently than RA in HepG2 cells, although the concentrations used were extremely high (25 μM) [225].

In conclusion, the treatment of HCC with synthetic retinoids warrants further research both in pre-clinical and clinical studies. The development of new, synthetic retinoids presents substantial advantages compared to RA when used in patients with HCC and may provide more efficient therapies for this deadly cancer.

## 7. General Summary

In summary, many studies have demonstrated that endogenous hepatic retinoid storage and metabolism are altered in all of the liver diseases discussed here. Moreover, numerous reports of therapeutic effects of RA and synthetic retinoids in these liver diseases have been published. The complexity of the retinoid signaling system provides a challenge to the identification of therapeutics, but our improved understanding of this complexity, coupled with the use of genetics and selective agonists and antagonists, holds great promise for the development of retinoid-based therapies for a wide variety of liver disorders where current therapies are not adequate.
